# SNPs Identified as Modulators of ECG Traits in the General Population Do Not Markedly Affect ECG Traits during Acute Myocardial Infarction nor Ventricular Fibrillation Risk in This Condition

**DOI:** 10.1371/journal.pone.0057216

**Published:** 2013-02-20

**Authors:** Raha Pazoki, Jonas S.S.G. de Jong, Roos F. Marsman, Nienke Bruinsma, Lukas R. C. Dekker, Arthur A. M. Wilde, Connie R. Bezzina, Michael W. T. Tanck

**Affiliations:** 1 Department of Clinical Epidemiology, Biostatistics & Bioinformatics, Academic Medical Center, Amsterdam, The Netherlands; 2 Department of Clinical and Experimental Cardiology, Heart Failure Research Center, Academic Medical Center, Amsterdam, The Netherlands; 3 Department of Cardiology, Catharina Hospital, Eindhoven, The Netherlands; Leibniz-Institute for Arteriosclerosis Research at the University Muenster, Germany

## Abstract

**Background:**

Ventricular fibrillation (VF) in the setting of acute ST elevation myocardial infarction (STEMI) is a leading cause of mortality. Although the risk of VF has a genetic component, the underlying genetic factors are largely unknown. Since heart rate and ECG intervals of conduction and repolarization during acute STEMI differ between patients who do and patients who do not develop VF, we investigated whether SNPs known to modulate these ECG indices in the general population also impact on the respective ECG indices during STEMI and on the risk of VF.

**Methods and Results:**

The study population consisted of participants of the Arrhythmia Genetics in the NEtherlandS (AGNES) study, which enrols patients with a first STEMI that develop VF (cases) and patients that do not develop VF (controls). SNPs known to impact on RR interval, PR interval, QRS duration or QTc interval in the general population were tested for effects on the respective STEMI ECG indices (stage 1). Only those showing a (suggestive) significant association were tested for association with VF (stage 2). On average, VF cases had a shorter RR and a longer QTc interval compared to non-VF controls. Eight SNPs showed a trend for association with the respective STEMI ECG indices. Of these, three were also suggestively associated with VF.

**Conclusions:**

RR interval and ECG indices of conduction and repolarization during acute STEMI differ between patients who develop VF and patients who do not. Although the effects of the SNPs on ECG indices during an acute STEMI seem to be similar in magnitude and direction as those found in the general population, the effects, at least in isolation, are too small to explain the differences in ECGs between cases and controls and to determine risk of VF.

## Introduction

Sudden cardiac death (SCD) is a leading cause of death in adults in the Western world [1]. The overwhelming majority (∼80%) of SCDs in adults are caused by the sequelae of coronary artery disease, namely myocardial ischemia or acute myocardial infarction (MI) [2]. While the risk of VF is known to have a genetic component, the underlying genetic factors are yet largely unknown [3]–[5].

Electrocardiographic indices of conduction and repolarization are considered important quantifiable intermediate phenotypes for arrhythmia risk [6]. In support of this concept, ECG indices measured during the acute phase of ST elevation MI (STEMI) were associated with risk of ensuing VF [7]. A number of studies have demonstrated that heart rate, PR interval, QRS duration and QTc interval are heritable traits [8]–[10] and over the last years genome-wide association studies (GWAS) in large samples of the general population have uncovered several common single nucleotide polymorphisms (SNPs) affecting these ECG traits [11]–[21].

The objective of this study was to assess whether SNPs, previously associated with heart rate and ECG indices of conduction and repolarization in GWAS in the general population, modulate ECG indices and risk of VF during acute STEMI. Specifically, we hypothesized that ECG-related SNPs modulate heart rate and ECG conduction and repolarization intervals in the setting of acute STEMI and thereby modulate (and predict) the risk of VF in this condition.

## Methods

### Ethics statement

The study protocol was approved by the Institutional Review Board of the Academic Medical Center, University of Amsterdam, and was conducted according to the principles of the Declaration of Helsinki. All participants gave written informed consent.

### Study samples

The study population consisted of participants of the **A**rrhythmia **G**enetics in the **NE**therland**S** (AGNES) case-control study which has been described in detail previously [3], [4]. In brief, the AGNES study enrols patients with a first acute STEMI. Cases are defined as patients who were successfully resuscitated after documented VF that occurred between the onset of symptoms and coronary intervention. Controls are defined as acute STEMI patients who did not develop VF. Patients with a previous MI or major co-morbidity were excluded. All individuals studied were of self-declared European ancestry.

### Measurement of ECG indices

The first recorded ECG that was acquired during STEMI and before reperfusion treatment was used for analysis. For cases, both ECGs acquired pre-VF as well as post-VF were included. ECGs of insufficient quality (due to e.g. baseline drift or missing leads) and ECGs with rhythms other than sinus rhythm or atrial fibrillation (AF) were excluded. Patients with AF (n = 23) were excluded for the analyses of PR interval, but included for the other ECG indices. All ECGs were digitized at 400 dpi (giving a spatial resolution 0.064 mm or 1.6 ms / pixel on an ECG traced at 25 mm / sec). Calibrated measurements were performed on-screen after 4 times enlargement of the digitized ECGs in ImageJ (National Institutes of Health, Bethesda, Maryland, http://rsb.info.nih.gov/ij/). RR-interval (heart rate) was measured from the ECG. PR interval was measured from the onset of the P-wave to the onset of ventricular depolarization. QRS duration was measured from the onset of ventricular depolarization to the J point. QTc interval was measured using the tangent method [22]. Lead II was used when the T wave was of sufficient amplitude to warrant QT measurement, otherwise leads V5 or V2 were used. QT was corrected for heart rate (QTc) using Fridericia's formula (QTc  =  QT / (cube root (RR)). ST deviation was calculated as the sum of all ST deviation from baseline at 60 ms after the J point in all 12 leads. ST deviation was not reported if individual leads were disconnected or in patients with left bundle branch block. The mean of three consecutive beats wherever possible was measured for all parameters and used in subsequent analyses. Patients with AV block, PR interval ≥ 200 ms or QRS duration ≥ 120 ms were excluded from the ECG analyses involving the continuous endpoints PR interval, QRS duration and QTc interval. For PR interval and QRS duration, additional dichotomous endpoints were assessed i.e. PR interval ≥ 200 ms and QRS duration ≥ 120 ms. Patients with AV block were included in the dichotomized PR interval endpoint as PR > 200 ms.

### Selection of SNPs and Genotyping

We inspected the published GWAS concerning heart rate and ECG indices of conduction and repolarization and identified SNPs reported to be associated with these parameters at genome-wide significant *P*-values (*P*<5×10^−8^) [23]. In case of high linkage disequilibrium (r2≥0.75) between identified SNPs, only a single SNP, capturing the maximum amount of variation present in the correlated SNPs, was selected for our analysis. This was the case for SNPs at the *SCN10A*, *SCN5A*, *KCNH2*, *KCNQ1*, *CNOT1*, *ARHGAP24*, *NOS1AP*, *CDKN1A, GJA1* and *MYH6* loci. A total of 65 SNPs were identified in this way. Genotypes for these SNPs were either obtained by direct genotyping (Illumina610 Quad genotyping array) or were estimated by imputation using HapMap build 36 as the reference panel. Details on genotyping and imputation in the AGNES sample have been described previously [4].

### Statistical Analysis

We tested differences between cases and controls in continuous variables with an independent sample t-test, or the Mann-Whitney U test when the data was not normally distributed. We compared differences in the percentages of categorical variables with the Pearson χ^2^ test. We used linear regression modelling in association analyses of continuous endpoints and logistic regression modelling for association analyses of dichotomous endpoints (VF, PR≥200 ms and QRS≥120 ms). In all models, we assumed an additive genetic model and corrected for age, sex and the culprit artery (harbouring the occluding lesion) [7] as well as the possible interaction between culprit artery and the SNPs. The occurrence of the latter was first tested using a Wald test.

Our analysis plan had a two-stage design. In the first stage, we tested for association of the selected SNPs with the corresponding ECG parameter during STEMI. In the second stage, we selected those SNPs with a (suggestive) significant effect on the ECG parameter and analyzed their effect on the occurrence of VF.

The Bonferroni thresholds for statistical significance were *P*≤0.0002 for the first stage (corrected for two tests per SNP and six outcomes: HR, QTc, PQ, PQ≥200, QRS and QRS≥120, resulting in a total of 210 tests) and *P*≤(0.05/number of SNPs carried over from stage 1) for the second stage. For both stages, *P*-values between the Bonferroni threshold and 0.05 were considered as a suggestive trend.

### Power and detectable effects

Given the observed standard deviations in our study population for heart rate and the ECG indices, the reported effects of the identified SNPs would result in effect sizes ≤ 0.2 SD. With the present range in sample sizes (417 – 515), the power to detect an effect size of 0.2 of a SNP with a minor allele frequency (MAF) ranging from 0.05 to 0.5 would range from 1 to 31% for a two-sided p-value of 0.0002 (Bonferroni threshold) and from 24 – 90% for a two-sided p-value of 0.05 (suggestive trend). The present study, therefore, lacks the power to significantly detect effect sizes as found in the general population (from the GWAs). However, given the fact that our heart rate and ECG indices were measured in patients experiencing a STEMI, we hypothesized that the SNP effects might be markedly increased in this sensitized population.

Given the present range in sample sizes, the study had 80% power to detect effect sizes of 0.7 to 0.3 (± 5% explained variance) for the quantitative traits at MAFs ranging from 0.05 to 0.5 assuming an additive genetic model and a two-sided significance threshold of 0.0002 (Stage 1). For PR interval with an overall standard deviation of 20 ms, for example, this translates into detectable allele effects (beta) ranging from 14 to 6 ms. For the dichotomized endpoints, we were able to detect odds ratio's ranging from 2.3 to 1.6 (Stage 1, α = 0.0002).

## Results

### VF and ischemic ECG indices

Baseline characteristics of the study population are shown in **Table 1**. As reported previously [3], [4], AGNES cases had a lower prevalence of diabetes mellitus and hypercholesterolemia, and lower mean body mass index (BMI) than AGNES controls. AGNES cases more often had a family history of sudden cardiac death as compared to controls.

**Table 1:** Baseline characteristics of the AGNES case-control set.

Characteristic	N*	Total	Cases	Controls	*P* ^‡^
		(n = 969)^†^	(n = 516)	(n = 453)	
Sex (male)		783 (80.6)	412 (80.0)	371 (81.2)	0.685
Mean age at myocardial infarction		56.4±11	55.9±11.2	56.9± 10.8	0.174
Family history of sudden death	909, 459, 450	291 (32.0)	174 (37.9)	117 (26.0)	<0.0001
CVD risk factors					
Current smoking	914, 473, 441	565 (61.8)	303 (64.1)	262 (59.4)	0.1531
Diabetes mellitus	893, 462, 431	62 (6.9)	21 (4.6)	41 (9.5)	0.004
Hypertension		294 (30.2)	149 (28.9)	145 (31.7)	0.3633
Hypercholesterolemia		297 (30.6)	127 (24.7)	170 (37.2)	<0.0001
Body mass index (BMI, kg/m^2^)	879, 439, 440	26.5±3.9	26.1±3.8	26.9±4.0	0.0042
Peak CK-MB	735, 337, 398	227 [294]	241 [354]	215 [247]	0.006
Ischemic ECG					
RR interval (ms)	453, 214, 239	807±204	743±188	864±203	<0.0001
ST segment deviation (mm)	474, 188, 286	15 [17]	20 [20]	13 [12]	<0.0001
PR interval (ms)	417, 195, 222	157±20	158±19	157±20	0.358
QRS duration (ms)	452, 214, 238	93±13	93±14	93±13	0.860
QTc interval (ms)	424, 200, 224	410±35	415±37	405±31	0.003
PR≥200 ms	515, 249, 266	57 (11.0)	32 (12.7)	25 (9.5)	0.153
QRS≥120 ms	509, 252, 257	57 (11.2)	38 (15.1)	19 (7.4)	0.007
Culprit artery	811, 430, 381				
RCA		220 (27.1)	109 (25.3)	111 (29.1)	
LAD		475 (58.6)	260 (60.5)	215 (56.4)	0.202
LCX		116 (14.3)	61 (14.2)	55 (14.4)	

CK-MB, creatine kinase-MB; LAD, left anterior descending artery; LCX, left circumflex artery; RCA, right coronary artery. *In case of missing values, the sample sizes of the total, case and control sets (total, case, control) for which information was available are given. ^†^ Normally distributed continuous variables are presented as mean ± SD or as Median [interquartile range] otherwise. Categorical variables data are presented as number (%). ‡ *P* value for comparison of cases and controls using independent t-test, Mann-Whitney test, or chi-square test where appropriate.

STEMI ECGs of sufficient quality were available for 599 patients. Of these, 79 (13%) patients had QRS≥120 ms, and 85 (14%) patients had a PR≥200 ms or higher degree AV block; several patients had a combination of these ECG abnormalities. Because of these exclusions and missing values, the number of available observations varied between 417 and 515. VF cases showed, on average, a shorter RR interval, a longer QTc interval, a greater ST segment deviation and more often prolongation of the QRS interval(≥120 ms) as compared to controls (**Table 1**). Location of the culprit coronary lesion modified the effect of QRS duration and QTc interval on risk of VF (**Table 2**). For instance, QRS duration was more prolonged in cases compared to controls only among patients with a left circumflex artery (LCX) occlusion (*P*_interaction_ = 0.002). QTc-interval tended to be more prolonged among patients with left anterior descending artery (LAD) or LCX occlusion (*P*_interaction_ = 0.026).

**Table 2:** ECG characteristics of AGNES cases and controls according to the artery harbouring the stenotic lesion.

	Culprit artery	Cases (N)	Mean±SD	Controls (N)	Mean±SD	*P* value*	Interaction *P* value
RR interval	LAD	130	728±170	124	822±180	<0.0001	
	LCX	25	751±185	35	866±213	0.047	0.974
	RCA	43	791±230	70	944±214	0.001	
PR interval	LAD	117	158±19	112	159±18	0.879	
	LCX	24	150±17	33	151±19	0.647	0.423
	RCA	39	163±20	67	157±22	0.168	
QRS duration	LAD	130	93±13	123	91±13	0.288	
	LCX	25	98±11	35	88±9	0.002	0.002
	RCA	43	94±16	70	98±12	0.134	
QTc interval	LAD	120	422±37	113	413±28	0.036	
	LCX	24	415±28	33	391±30	0.020	0.026
	RCA	40	399±38	68	399±35	0.939	

LAD, left anterior descending artery; LCX, left circumflex artery; RCA, right coronary artery

* *P* value of comparison between cases and controls using a logistic regression model adjusted for age and sex. (All patients with AV block or PR≥200 ms or QRS≥120 ms & AF are excluded)

### ECG candidate SNPs and STEMI ECG indices (Stage 1)

None of the 65 SNPs tested displayed an association with the corresponding ECG trait that passed the Bonferroni-corrected significance threshold of *P*≤0.0002, nor showed an interaction with culprit artery. Eight SNPs displayed a trend (0.0002<*P*<0.05) in association with STEMI ECG indices (**Table 3**). Regarding the continuous ECG outcomes, SNPs rs223116 and rs281868 showed a trend for association with RR interval (*P* = 0.004 and 0.014, respectively) and SNP rs6795970 showed a trend for association with PR interval (*P* = 0.004). SNPs rs1886512 and rs883079 showed a trend for association with QRS duration (*P* = 0.010 and 0.007, respectively). SNPs rs17779747 and rs8049607 showed a trend for association with QTc interval (*P* = 0.004 and 0.036, respectively). Regarding the dichotomized ECG endpoints, the C-allele of SNP rs11708996 showed a trend for association with PR≥200 ms with an odds ratio of 2.39 (95% CI: 1.33 – 4.30; *P* = 0.004). None of the QRS SNPs showed any significant association with QRS≥120 ms.

**Table 3:** Association analysis of SNPs with ECG indices of conduction and repolarization during myocardial ischemia.

SNP	End point	Coded/Non Coded Allele	GWAS Effect^*^	Minor Allele (Frequency)	Beta (SE)^†^	*P* value	*P* value Interaction	Gene
RR SNPs								
rs11154022	RR	A/G	Inc	A (0.33)	6.5 (14.9)	0.664	0.975	*8 kb from GJA1*
rs12666989	RR	C/G	Dec	C (0.20)	−10.7 (17.6)	0.543	0.175	*UFSP1*
rs12731740	RR	T/C	Inc	T (0.12)	10.3 (22.3)	0.646	0.277	*CD3-MIR29C-MIR29B2*
rs17287293	RR	G/A	Inc	G (0.14)	22.6 (19.7)	0.273	0.128	*SOX5-BCAT1*
rs174547	RR	C/T	Dec	C (0.31)	−11.7 (15.0)	0.438	0.343	*FADS1*
rs223116	RR	A/G	Dec	A (0.24)	−45.6 (15.9)	0.004	0.461	*THTPA-NGDN-ZFHX2-MYH7*
rs2745967	RR	G/A	Inc	G (0.38)	−1.2 (14.1)	0.932	0.168	*CD34-PLXNA2*
rs281868	RR	G/A	Dec	A (0.49)	−33.8 (13.8)	0.014	0.061	*SLC35F1*
rs314370	RR	C/T	Dec	C (0.20)	−5.5 (17.4)	0.751	0.197	*SLC12A9*
rs452036	RR	A/G	Dec	A (0.39)	−10.4 (14.5)	0.472	0.823	*MYH6*
rs885389	RR	A/G	Dec	A (0.37)	−17.2 (14.7)	0.243	0.571	*GPR133*
rs9398652	RR	A/C	Dec	A (0.12)	−9.2 (21.9)	0.674	0.292	*GJA1-HSF2*
PR SNPs								
rs11047543	PR	A/G	Dec	A (0.14)	−1.97 (1.94)	0.312	0.696	*SOX5*
rs11708996	PR	C/G	Inc	C (0.16)	−0.64 (2.11)	0.763	0.722	*SCN5A*
rs11897119	PR	C/T	Inc	C (0.38)	0.41 (1.42)	0.775	0.407	*MEIS1*
rs1896312	PR	C/T	Inc	C (0.29)	0.37 (1.49)	0.807	0.399	*TBX5-TBX3*
rs251253	PR	C/T	Dec	C (0.41)	−1.59 (1.37)	0.248	0.744	*NKX2-5*
rs3807989	PR	A/G	Inc	A (0.42)	0.17 (1.42)	0.904	0.170	*CAV1-CAV2*
rs3825214	PR	G/A	Inc	G (0.20)	−0.16 (1.74)	0.925	0.766	*TBX5*
rs4944092	PR	G/A	Dec	G (0.33)	−0.74 (1.48)	0.616	0.046	*WNT11*
rs6795970	PR	A/G	Inc	A (0.39)	4.06 (1.42)	0.004	0.031	*SCN10A*
rs7660702	PR	T/C	Inc	C (0.30)	−0.04 (1.42)	0.979	0.462	*ARHGAP24*
QRS SNPs								
rs10850409	QRS	A/G	Dec	A (0.25)	−0.39 (0.98)	0.696	0.380	*TBX3*
rs10865879	QRS	T/C	Inc	C (0.25)	−0.45 (1.07)	0.671	0.792	*SCN5A/EXOG*
rs11153730	QRS	C/T	Inc	T (0.49)	−0.72 (0.87)	0.408	0.383	*C6orf204-SLC35F1-PLN-BRD7P3*
rs11708996	QRS	C/G	Inc	C (0.16)	0.94 (1.37)	0.493	0.232	*SCN5A*
rs11710077	QRS	T/A	Dec	T (0.18)	0.59 (1.12)	0.596	0.517	*SCN5A*
rs11848785	QRS	G/A	Dec	G (0.27)	−0.34 (1.05)	0.743	0.756	*SIPA1L1*
rs13165478	QRS	A/G	Dec	A (0.37)	−1.45 (0.91)	0.115	0.559	*HAND1-SAP30L*
rs1321311	QRS	A/C	Inc	A (0.24)	−0.43 (1.08)	0.691	0.858	*CDKN1A*
rs1362212	QRS	A/G	Inc	A (0.16)	0.65 (1.29)	0.616	0.834	*TBX20*
rs17020136	QRS	C/T	Inc	C (0.21)	0.24 (1.08)	0.827	0.251	*HEATR5B-STRN*
rs1733724	QRS	A/G	Inc	A (0.27)	−0.51 (1.03)	0.621	0.274	*DKK1*
rs17391905	QRS	G/T	Dec	G (0.02)	−2.68 (3.13)	0.392	0.978	*C1orf185-RNF11-CDKN2C-FAF1*
rs17608766	QRS	C/T	Inc	C (0.13)	1.31 (1.31)	0.320	0.453	*GOSR2*
rs1886512	QRS	A/T	Dec	A (0.37)	−2.39 (0.92)	0.010	0.880	*KLF12*
rs2051211	QRS	G/A	Dec	G (0.26)	−0.35 (1.03)	0.734	0.271	*EXOG*
rs2242285	QRS	A/G	Inc	A (0.43)	−0.24 (0.87)	0.784	0.888	*LRIG1-SLC25A26*
rs4074536	QRS	C/T	Dec	C (0.32)	1.19 (0.99)	0.232	0.749	*CASQ2*
rs4687718	QRS	A/G	Dec	A (0.12)	1.70 (1.34)	0.207	0.487	*TKT-PRKCD-CACNA1D*
rs6795970	QRS	A/G	Inc	A (0.39)	0.52 (0.91)	0.569	0.326	*SCN10A*
rs7342028	QRS	T/G	Inc	T (0.25)	1.89 (1.03)	0.068	0.051	*VTI1A*
rs7562790	QRS	G/T	Inc	G (0.43)	−0.22 (0.91)	0.812	0.529	*CRIM1*
rs7784776	QRS	G/A	Inc	G (0.41)	−0.82 (0.93)	0.383	0.265	*IGFBP3*
rs883079	QRS	C/T	Inc	C (0.26)	2.77 (1.02)	0.007	0.147	*TBX5*
rs9436640	QRS	G/T	Dec	G (0.48)	−0.68 (0.89)	0.445	0.278	*NFIA*
QRS SNPs								
rs9851724	QRS	C/T	Dec	C (0.35)	−1.25 (0.92)	0.177	0.527	*SCN10A*
rs991014	QRS	T/C	Inc	T (0.43)	−0.40 (0.91)	0.665	0.427	*SETBP1*
rs9912468	QRS	G/C	Inc	G (0.40)	0.28 (0.96)	0.770	0.511	*PRKCA*
QTc SNPs								
rs10919071	QTc	A/G	Inc	G (0.11)	−0.01 (2.09)	0.997	0.774	*ATP1B1*
rs11970286	QTc	T/C	Inc	T (0.47)	0.13 (2.31)	0.954	0.996	*PLN*
rs12029454	QTc	A/G	Inc	A (0.14)	4.74 (3.20)	0.139	0.439	*NOS1AP*
rs12053903	QTc	C/T	Dec	C (0.33)	−4.13 (2.55)	0.106	0.911	*SCN5A*
rs12143842	QTc	T/C	Inc	T (0.24)	3.47 (2.69)	0.197	0.874	*NOS1AP*
rs12210810	QTc	C/G	Dec	C (0.04)	1.34 (5.33)	0.801	0.590	*PLN*
rs12296050	QTc	T/C	Inc	T (0.18)	0.19 (2.98)	0.948	0.914	*KCNQ1*
rs16857031	QTc	G/C	Inc	G (0.14)	6.18 (3.28)	0.060	0.571	*NOS1AP*
rs17779747	QTc	T/G	Dec	T (0.34)	−7.36 (2.52)	0.004	0.811	*KCNJ2*
rs1805128	QTc	A/G	Inc	T (0.05)	8.69 (5.56)	0.119	0.509	*KCNE1*
rs2074238	QTc	T/C	Dec	T (0.05)	−3.30 (6.68)	0.622	0.136	*KCNQ1*
rs2074518	QTc	T/C	Dec	T (0.49)	−1.13 (2.37)	0.633	0.683	*LIG3*
rs2968863	QTc	T/C	Dec	T (0.26)	−1.93 (2.84)	0.499	0.266	*KCNH2*
rs37062	QTc	G/A	Dec	G (0.23)	2.25 (2.84)	0.429	0.359	*CNOT1*
rs4657178	QTc	T/C	Inc	T (0.25)	3.78 (2.71)	0.165	0.543	*NOS1AP*
rs4725982	QTc	T/C	Inc	T (0.19)	0.71 (3.11)	0.819	0.674	*KCNH2*
rs8049607	QTc	T/C	Inc	C (0.49)	5.02 (2.39)	0.036	0.145	*LITAF*
rs846111	QTc	C/C	Inc	C (0.29)	0.70 (2.84)	0.806	0.056	*RNF207*

SE, Standard Error * Direction of effect estimate per copy coded allele; Inc, Increasing effect; Dec, Decreasing effect; data from previous GWA studies † Effect estimate is given per copy of the coded allele adjusted for age, sex and culprit artery (all patients with AV block or PR ≥ 200 ms or QRS ≥ 120 ms are excluded).

### STEMI ECG SNPs and risk of VF (Stage 2)

We next tested the eight SNPs from Stage 1 showing a trend for association with the ECG parameters for association with VF. Of these eight, none passed the Bonferroni-corrected significance threshold (*P*≤0.0063) for association with risk of VF during STEMI. However, two SNPs, namely rs6795970 and rs17779747, were nominally associated with VF (*P* = 0.009 and 0.026, respectively) independent of the culprit artery. Another SNP, rs223116 displayed a nominal association with VF in patients with an occlusion in the LCX (*P* = 0.039) or RCA (*P* = 0.037) only (**Table 4**).

**Table 4:** Association analysis of SNPs with VF in AGNES cases versus AGNES controls.

SNP	GWAS End point	Coded/Non Coded Allele	GWAS Effect*	Minor Allele (Frequency)	Odds ratio [95% CI]*	*P* value	*P* value Interaction^†^	Gene
rs223116	RR	A/G	Dec	A (0.24)				*THTPA-NGDN-*
LAD					0.86 [0.64 – 1.17]	0.340	0.0163	*ZFHX-MYH7*
LCX					2.08 [1.04 – 4.17]	0.039		
RCA					1.61 [1.03 – 2.51]	0.037		
rs281868	RR	G/A	Dec	A (0.49)	0.99 [0.81 – 1.21]	0.929	0.237	*SLC35F1*
rs6795970	PR	A/G	Inc	A (0.39)	0.77 [0.63 – 0.94]	0.009	0.897	*SCN10A*
rs11708996	PR≥200 ms	C/G	Inc	C (0.16)	1.24 [0.93 – 1.65]	0.144	0.235	*SCN5A*
rs1886512	QRS	A/T	Dec	A (0.37)	0.98 [0.80 – 1.20]	0.824	0.549	*KLF12*
rs883079	QRS	C/T	Inc	C (0.26)	0.90 [0.72 – 1.13]	0.359	0.819	*TBX5*
rs17779747	QTc	T/G	Dec	T (0.34)	1.27 [1.03 – 1.57]	0.026	0.921	*KCNJ2*
rs8049607	QTc	T/C	Inc	C (0.49)	1.05 [0.87 – 1.29]	0.597	0.942	*LITAF*

* effect estimate is given per copy of the coded allele adjusted for age, sex and culprit artery. † *P* values for interaction between SNPs and culprit artery on risk of VF

## Discussion

In the current study, we confirm and extend previous observations that heart rate and ECG indices of conduction and repolarization differ between acute STEMI patients who develop VF and acute STEMI patients who do not develop VF. Although our analysis only detected nominal association with the STEMI ECG traits for only a minority of SNPs, the effect size and direction for the association of these SNPs was comparable to that found for the corresponding trait in the general population. Furthermore, of the eight SNPs displaying such association, three also displayed a trend for association with VF.

### Ischemic ECG indices and VF

Ischemic ECGs of patients with VF (cases) showed, on average, shorter RR intervals, longer QTc intervals and more ST segment deviation than patients without VF (controls). When the culprit artery harbouring the occluding lesion was taken into account, cases with an LCX occlusion had a longer QRS duration. This finding is similar to our previous findings in a smaller sample of STEMI patients with and without VF [7]. However, the observation in this previous study of longer QRS interval in cases with an RCA occlusion was not observed in the present study. With respect to QTc interval, cases with either an LAD or LCX occlusion had a longer QTc-interval compared to controls. This extends our earlier findings, where similar (though non-significant) differences in QTc interval were found for the same culprit arteries. The observed longer QRS duration and QTc interval in cases with VF reflect cardiac conduction delay and prolonged repolarization time, respectively, consistent with a more pro-arrhythmic substrate in cases [24].

### ECG candidate SNPs, STEMI ECG indices and VF

Recent GWAS have identified multiple common genetic variants affecting heart rate, conduction and repolarization in individuals from the general population [11]–[21]. However, whether these variants also influence ECG indices in the setting of STEMI has not yet been investigated. In our study, none of the association P-values exceeded our stringent pre-set threshold for statistical significance. Eight SNPs displayed a suggestive association to the corresponding STEMI ECG parameter, and of note all eight SNPs displayed an effect that was similar in direction and magnitude to that observed in the general population [11]–[21]. However our findings also demonstrate that SNPs which are known to exert only small effects on ECG parameters in the general population, do not have a markedly increased effect on ECG indices in the setting of acute STEMI.

None of the eight SNPs that displayed nominal association with STEMI ECG parameters passed our stage 2 Bonferroni threshold (*P*<0.0063) for the association with VF. However, as previously reported [11], the A-allele of rs6795970 in the *SCN10A* gene appears to be associated with a decreased risk of VF in the AGNES population (*P* = 0.009). The rs6795970 SNP has consistently been shown to impact on PR interval in different studies carried out in the general population [11]–[13], with the A-allele being the PR-prolonging allele. We now show that this SNP may also be associated with a longer PR interval during acute STEMI. In addition to PR interval, rs6795970 has been found associated with QRS in GWA studies, underscoring the shared molecular underpinning of atrio-ventricular and ventricular conduction. In spite of this, the effect of rs6795970 on QRS duration in our study was small and non-significant. SNP rs6795970 is in high LD (r^2^ = 0.93) with rs6801957 with both SNPs being localized within the *SCN10A* gene located immediately downstream of *SCN5A* on chromosome 3. SNP rs6801957 is located in a T-box transcription factor binding motif within an enhancer element and the PR- and QRS-prolonging minor allele of this SNP was recently shown to affect TBX3/TBX5 binding and T-box factor response of the enhancer [25]. These recent findings suggest that rs6801957 is the causal SNP and that this SNP may modulate *SCN5A* and / or *SCN10A* expression levels in human heart and thereby impact on conduction and arrhythmia risk.

The common genetic variants identified to date as modulators of ECG indices in the general population all carry very small effect sizes and even when considered in aggregate, explain only a very small percentage of the variation in these indices. For instance, in a meta-analysis for identification of QTc-associating SNPs by Pfeufer and co-workers [15], SNPs at 10 different loci in aggregate explained only around 3% of the variance in this trait. The effects of these common genetic variants on the STEMI ECG are small and insufficient to explain the differences found in STEMI ECGs of cases and controls. This underscores the need for further studies aimed at uncovering additional genetic variants, such as rare variants associated with larger effects, which would lead to a more-complete representation of the allelic architecture of these differences in STEMI ECGs.

### Strengths and Limitations

In this study, we had the unique opportunity to study the effect of genetic variants, known to impact on ECG indices in the general population, on VF in a well-defined case-control set of patients with a first acute STEMI. Our study is rather unique in that the availability of STEMI ECGs and DNA from patients in whom STEMI is complicated by VF is very scarce due to the high mortality in these patients. On the other hand, as a consequence, our sample size is limited, which can result in insufficient statistical power. Furthermore, the STEMI ECGs used in this study were retrieved retrospectively and, therefore, the time between the onset of complaints and acquisition of the STEMI ECG varied among the study participants. Also since the ECGs were retrieved retrospectively, some were of insufficient quality for analysis, limiting the size of the sample available for analysis. The first recorded ECG that was acquired during STEMI and before reperfusion treatment was used for this analysis. Due to the nature of this complex and specific phenotype under investigation a resting ECG without STEMI prior to the index event is missing for most of the patients and the effects of SNPs on baseline ECGs from these patients could, therefore, not be tested. Our analysis did not account for effects which may arise from differences in infarct size, intake of amiodarone or cardioversion. However, with respect to infarct size, additional adjustment for peak CKMB as a marker of infarct size, resulted in similar effect estimates but with larger confidence intervals, mainly related to the reduced sample size as peak CKMB was not available in all patients (**Table S1**).

## Conclusion

In conclusion, ECG indices of conduction and repolarization differ between STEMI patients with VF and STEMI patients who do not develop VF. Although the effects of some SNPs on ECG parameters during an acute STEMI were similar in magnitude and direction as those found in the general population, the effects were too small to explain the differences in conduction and repolarization indices and to exert any marked impact on risk of VF. Nevertheless, rs6795970, located within the *SCN10A* gene, associated with longer PR interval in the general population and with longer PR interval and risk of VF during STEMI in our study, merits investigation in future larger studies.

## Supporting Information

Table S1 **Association analysis of SNPs with VF in AGNES cases versus AGNES controls with additional correction for peak CKMB levels.**
(DOC)
