# Supplementary material for: SNPs Identified as Modulators of ECG Traits in the General Population Do Not Markedly Affect ECG Traits during Acute Myocardial Infarction nor Ventricular Fibrillation Risk in This Condition
Source: PLoS One. 2013 Feb 20;8(2):e57216. doi: 10.1371/journal.pone.0057216 (PMC3577709; doi:10.1371/journal.pone.0057216)
Supplement: Table S1 — Association analysis of SNPs with VF in AGNES cases versus AGNES controls with additional correction for peak CKMB levels. (DOC) [file pone.0057216.s001.doc]

**Table S**1. Association analysis of SNPs with VF in AGNES cases versus AGNES controls with additional correction for peak CKMB levels.

| **SNP** | **GWAS End point** | **Coded/Non Coded Allele** | **GWAS Effect^*^** | **Minor Allele (Frequency)** | **Odds ratio [95% CI]^*^** | ***P* value** | ***P* value Interaction^†^** | **Gene** |
| --- | --- | --- | --- | --- | --- | --- | --- | --- |
| rs223116 | RR | A/G | Dec | A (0.24) | 1.04 [0.79 – 1.36] | 0.785 | 0.286 | *THTPA – NGDN – ZFHX2* |
| rs281868 | RR | G/A | Dec | A (0.49) | 0.95 [0.76 – 1.19] | 0.650 | 0.237 | *SLC35F1* |
| rs6795970 | PR | A/G | Inc | A (0.39) | 0.81 [0.64 – 1.02] | 0.078 | 0.696 | *SCN10A* |
| rs11708996 | PR ≥ 200 ms | C/G | Inc | C (0.16) | 1.26 [0.91 – 1.77] | 0.169 | 0.118 | *SCN5A* |
| rs1886512 | QRS | A/T | Dec | A (0.37) | 0.93 [0.74 – 1.18] | 0.549 | 0.153 | *KLF12* |
| rs883079 | QRS | C/T | Inc | C (0.26) | 1.02 [0.79 – 1.31] | 0.900 | 0.836 | *TBX5* |
| rs17779747 | QTc | T/G | Dec | T (0.34) | 1.15 [0.90 – 1.47] | 0.263 | 0.644 | *KCNJ2* |
| rs8049607 | QTc | T/C | Inc | C (0.49) | 0.97 [0.77 – 1.22] | 0.780 | 0.941 | *LITAF* |

*effect estimate is given per copy of the coded allele adjusted for age, sex, culprit artery and CKMB. † *P* values for interaction between SNPs and culprit artery on risk of VF
